# Morphology and Histological Observation of the Male Reproductive System in the Swimming Crab (*Portunus trituberculatus*)

**DOI:** 10.3390/biology14121697

**Published:** 2025-11-28

**Authors:** Hao Wang, Nahayo Viateur, Litao Wan, Peng Tan, Jie He, Lijian Xue

**Affiliations:** 1 College of Fisheries, Zhejiang Ocean University, Zhoushan 316022, China; 17802520096@163.com (H.W.); nahayoviateur04@gmail.com (N.V.); 2 Experimental Base of Zhejiang Marine Fisheries Research Institute, Zhoushan 316021, China; wan_066934@126.com (L.W.); tanpeng@zjou.edu.cn (P.T.); he_0902@126.com (J.H.)

**Keywords:** *Portunus trituberculatus*, male gonopods, copulatory system, structure and function

## Abstract

The swimming crab (*Portunus trituberculatus*) is an economically important marine crab species in China. Understanding the structure and function of the male reproductive system in the swimming crab is crucial for increasing reproductive efficiency. Although studies on its reproductive biology have increased in recent years, the ultrastructure of the male copulatory system remains insufficiently explored. In this study, gonadal tissues [testes (T), anterior vas deferens (AVD), middle vas deferens (MVD), posterior vas deferens (PVD)] and copulatory organs [first gonopod (G1), second gonopod (G2), penis] of *P. trituberculatus* were sampled as experimental materials, and we systematically investigated the morphological and functional characteristics of male reproductive and copulatory organs in *P. trituberculatus* using anatomical, stereomicroscopic and scanning electron microscopic techniques. The results indicate that the male copulatory system comprises the G1, G2, and the penis. The G1 shows a tubular structure, with its endopodite bearing spines that anchor the female during copulation. The G2 is short, stout, solid and rod-shaped, with surface folds and blunt teeth, indicating its role in propelling the spermatophore within the G1. The penis is flexible and covered with elongated, rigid setae. Internally, the reproductive system displays a bilaterally symmetrical “H”-shaped architecture, consisting of the testes; anterior, middle, and posterior vas deferens; and the ejaculatory ducts. These segments have different structures and function in forming, storing, and transporting spermatophores. These findings provide important insights into the reproductive strategies and mating mechanisms of *P. trituberculatus*.

## Simple Summary

The swimming crab is a highly valuable seafood species in China, but how male crabs successfully mate and transfer sperm is not well understood. This study aimed to closely examine the detailed structure of the male crab’s reproductive organs to learn how they work together during mating. We found that the male uses three key parts: a long, hollow tube with spines to hold onto the female, a short, rod-like part that acts like a plunger to push the sperm forward, and a flexible penis covered with tiny hairs. Inside the body, the reproductive system is shaped like an “H” and includes different sections that produce, store, and deliver sperm packets. These discoveries help explain the mating process of the swimming crab and provide important knowledge to improve breeding methods in crab farming, supporting sustainable production and conservation of this valuable species.

## 1. Introduction

The male reproductive system in brachyuran crabs typically comprises an external copulatory apparatus, consisting of the penis, the first gonopod (G1), and the second gonopod (G2), along with internal paired structures, including the testes, vasa deferentia (VD), and ejaculatory ducts (ED). This organizational architecture is conserved across all brachyurans [1,2,3]. Furthermore, the morphology of the crab reproductive system is a product of long-term natural and sexual selection, reflecting its adaptive strategies to diverse ecological environments [4]. Within the Brachyura, advanced taxa exhibit several evolutionary trends in their copulatory apparatus. The G1 endopod is specialized into a tubular structure serving as a conduit for spermatophore transfer, whereas the G2 endopod) is significantly reduced and fused, functioning as a piston. The penis extends externally from the ejaculatory duct and connects to the basal opening of the G1; these three components function together to facilitate copulation [5,6,7].

The highly specialized morphology of the male reproductive system is a key taxonomic characteristic for identifying brachyuran species [8]. The morphological variation in the male copulatory apparatus across different species is closely correlated with their mating strategies. In some crab species, an elongated G1 endopod allows direct insertion into the female’s gonopore for spermatophore transfer. However, a columnar G1 endopod does not penetrate directly but instead forms an “abutting” or “coupling” type of copulatory system [9,10,11]. Moreover, the relative length of gonopods is critical for functional determination, where the G2 is considerably longer than the G1 and G2 can directly penetrate female gonopores. Conversely, in species where the G2 is shorter than the G1, sperm delivery to the female’s spermatheca relies on a pumping mechanism facilitated by the G2 [12,13].

The internal male reproductive system in brachyurans typically has a bilobed, “H”-shaped architecture within the cephalothoracic cavity [14,15]. They are connected posteriorly to the stomach and anteriorly to the heart by a transverse commissure [16,17]. The VD is typically subdivided into three distinct regions: the anterior vas deferens (AVD), the median vas deferens (MVD), and the posterior vas deferens (PVD). The AVD is the primary site for spermatophore formation, whereas the MVD and PVD collectively store mature spermatophores and semen [18]. Studies on blue land crab(*Cardisoma guanhumi*) (Latreille, 1828) and the ornate blue crab (*Callinectes ornatus*) (Ordway, 1863) have confirmed that the AVD is the core region for spermatophore formation [19,20], which provides a basis for understanding the common mechanisms of spermatophore development in brachyurans. The ejaculatory duct is a slender, smooth-walled tube that extends posteroventrally along the coxa of the fifth pereiopod, propelling spermatophores toward the gonopore via muscular contractions [21].

*P. trituberculatus* (Miers, 1876) is a highly valued commercial crab species in China. It has a broad distribution range along the coasts of China from north to south and extends to the offshore waters of Korea and Japan [22]. In 2024, the total national catch of swimming crabs reached 450,000 tons, and mariculture production reached 100,000 tons, making it a pillar species in China’s mariculture industry [23]. While existing research on *P. trituberculatus* reproductive biology has focused on female ovarian development and embryogenesis [24,25,26,27], investigations into the male reproductive system are relatively limited; specifically, the ultrastructure of the copulatory system has yet to be thoroughly described [28,29]. The ability of males to complete copulation, transfer spermatophores and influence the female fertilization rate is critically dependent on the morphological compatibility and functional integrity of their copulatory system. Hence, the mating strategies of *P. trituberculatus* remain poorly understood. To address this gap, the present study aims to provide a systematic elucidation of the ultrastructural and morpho-functional aspects of both the reproductive and copulatory systems in *P. trituberculatus*, utilizing stereomicroscopy and scanning electron microscopy (SEM).

## 2. Materials and Methods

### 2.1. Experimental Crabs

**Figure 1:**
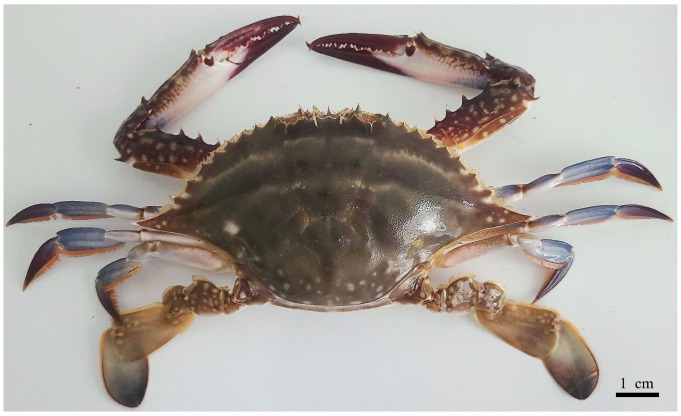
Overall Morphological Characteristics of Male Individuals of the Swimming Crab (*Portunus trituberculatus*).

This study was conducted in September 2024 at the experimental station of the Zhejiang Marine Fisheries Research Institute. Sexually mature, healthy male crabs with intact abdomens and no prior mating history were collected from all-male culture ponds at the station using baited traps. The body weight of the selected crabs was 200.0 ± 5.23 g (Figure 1). According to the study by Sun, Y. et al., male swimming crabs (*Portunus trituberculatus*) require 8–10 molts to develop from Stage I juvenile crabs to the sexually mature stage, and the body weight of mature individuals can reach 55.5–176.4 g [22]. All samples used in the experiment met the sexually mature criteria.

### 2.2. Stereomicroscopy

A total of six male crabs were dissected under ice-bath conditions to collect complete gonadal tissues (T, AVD, MVD, PVD) and copulatory organs (G1, G2). The gonadal tissues were directly examined under a stereomicroscope to observe their external and internal morphology. The copulatory organ samples were fixed in 95% ethanol for 24 h, after which their morphological structures were observed, photographed, and analyzed under a stereomicroscope.

### 2.3. Scanning Electron Microscopy (SEM)

Six male crabs were dissected under ice-bath conditions to harvest their copulatory organs (G1, G2, and penis) for examination under a scanning electron microscope (SEM, HITACHI, SU8100, Tokyo, Japan) to characterize their tissue architecture. The detailed procedures were as follows.

#### 2.3.1. Sample Pre-Treatment

The copulatory organs (G1, G2, and penis) were dissected free of excess tissue and immersed in glutaraldehyde electron microscopy fixation buffer (composed of 2.5% glutaraldehyde, with 0.1 M phosphate buffer as the solvent, pH 7.0–7.5, at 25 °C) to ensure complete tissue infiltration. Primary fixation was conducted in this glutaraldehyde buffer at 4 °C for 4 h, followed by three 15-min washes in 0.1 M phosphate-buffered saline (PBS). Subsequently, secondary fixation was performed using 1% osmium tetroxide in 0.1 M PBS for 2 h, after which the samples were washed three times with 0.1 M PBS (15 min each).

#### 2.3.2. Sample Preparation

The tissues were dehydrated through a graded ethanol series (50%, 70%, 80%, 90%, 95%, and 100%) for 15 min at each concentration. They were then infiltrated overnight with a 1:1 mixture of acetone and 812 embedding resin. Polymerization of the embedded samples was carried out at 60 °C for 48 h. Ultrathin sections (60–80 nm) were prepared using an ultramicrotome. The sections were subjected to double-staining with uranium and lead and were left to dry overnight at room temperature.

#### 2.3.3. SEM Observation and Analysis

The key structural regions of the male copulatory organs (G1 and G2) were located on the SEM viewing screen. Once identified, the focus and magnification were adjusted to optimize image clarity, and micrographs were captured for subsequent analysis, which was performed using Photoshop 2020.

### 2.4. Data Analysis

All experimental data (including the body weight data of male Portunus trituberculatus) were statistically analyzed using SPSS 29.0 software. Measurement data were expressed as (mean ± SD).

## 3. Results

### 3.1. Morphology and Function of External Copulatory Organs in the Reproductive System

#### 3.1.1. First Gonopod (G1)

**Figure 2:**
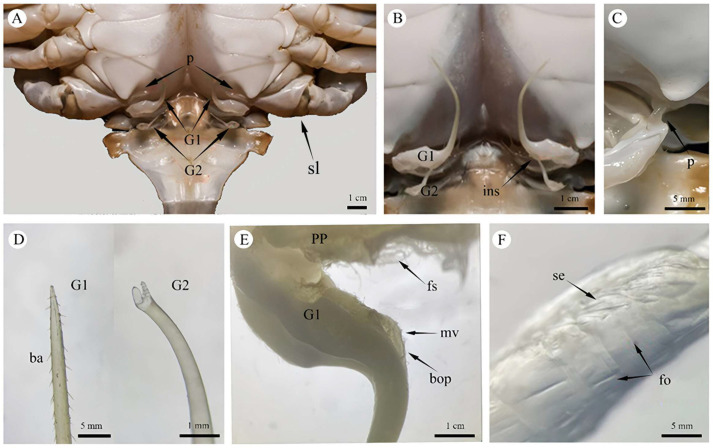
External Copulatory Organs of the Male *P*. *trituberculatus* Reproductive System. (**A**) Overall composition of the male copulatory system. (**B**) External morphology of the G1 and G2, showing the state in which G2 was inserted into G1. (**C**) Penis, showing the gonoporal opening and its inserted state within the G1 base. (**D**) Stereomicroscopic view of the upper structure of the G1 and G2. (**E**) Stereomicroscopic view of the basal opening of the G1 endopod, showing the entrance for the G2 and plumose setae. (**F**) Stereomicroscopic view of the penis, showing cuticular folds on the penile surface. G1: first gonopod; G2: second gonopod; p: penis; sl: swimming leg; ins: inserted state; ba: barb; pp: protopodite; mv: membranous valve; bop: basal opening of G1; fs: plumose setae; fo: folds; se: seta.

**Figure 3:**
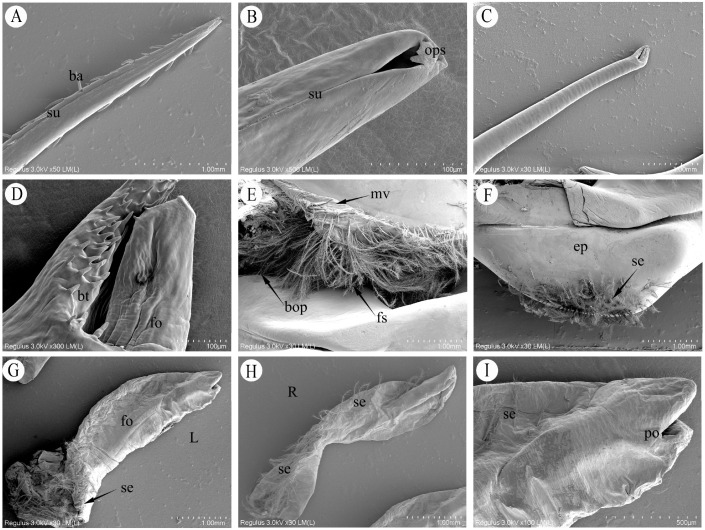
Scanning electron microscopy (SEM) of the male copulatory system of *P. trituberculatus*. (**A**) Upper structure of the G1, showing robust lateral barbs and longitudinal suture grooves. (**B**) Apex of the G1, showing the pinhole-like apical opening. (**C**) Upper structure of the second gonopod G2, showing the smooth tubular wall. (**D**) Fish-mouth-shaped apex of the G2, showing blunt teeth on the lower portion and surface folds on the upper portion. (**E**) Basal opening region of the G1, showing the entrance for the G2 and surrounding plumose setae. (**F**) Basal morphological structure of the G1 endopod. (**G**) Lateral view of the penis, showing its external morphology (L: lateral view). (**H**) Medial view of the penis, showing the ventral setae (R: ventral view). (**I**) Gonoporal opening of the penis.ba: barb; su: suture groove; ops: opening at the G1 apex; bt: blunt teeth; fo: folds; mv: membranous valve; bop: basal opening of G1; fs: plumose setae; ep: endopod; se: seta; po: penile opening.

The male copulatory system of *P. trituberculatus* consists of two pairs of uniramous external gonopods modified from male pleopods located on the first and second abdominal segments (Figure 2A). The first gonopod (G1) comprises a basal protopod and a slender endopod. The G1 is long, thin and slightly flattened dorsoventrally. It appears tubular, hollow and tapers distally. At the apex is a pinhole-like opening, which is formed by the infolding of the endopod cuticle (Figure 3B). Behind this opening, a suture groove runs along the length of the endopod. The lateral sides bear two rows of robust, downward-projecting spines, covering approximately one-third of the G1 length (Figure 2D and Figure 3A). The middle section of the G1 is smooth and straight. The distal end of the endopod curves strongly outward from the body. The base nearer the sternum is divided into two parts, and the opening nearer the sternum is covered by a valvular flap fringed with setae. During mating, the penis, which is located on the lateral surface at the base of the endopod, extends into this flap to transfer the spermatophore (Figure 2C). The medial opening is densely setose and serves as the insertion point for the G2 (Figure 2E and Figure 3E).

#### 3.1.2. Second Gonopod (G2)

The second gonopod (G2) was significantly smaller than G1, approximately one-fifth of its length (Figure 2B). While structurally similar to those of G1 (Figure 2D), the protopod and endopod of the G2 were almost entirely fused, with a discernible suture line remaining between them. The G2 presented as a solid, rod-like structure with a closed, fish-mouth-shaped apex. Its upper portion was columnar and covered with long, slit-like folds (Figure 3C,D), while the lower portion was slightly flattened, with the wall projecting outward to form numerous blunt teeth. The base of the protopod curved strongly outward, similar to that of the G1, and consisted of a flexible, membranous area densely surrounded by setae. Functionally, the G2 is thought to act as a piston or plunger within the lumen of the G1, propelling the sperm mass from the ejaculatory duct toward its distal opening.

#### 3.1.3. Penis

The distal portion of the VD, which is more muscular, is referred to as the ejaculatory duct. The penis represents the terminal extension of the ejaculatory duct, opening externally on the body surface at the gonopore. The penis was small and translucent, with an exposed length of 0.4–0.8 cm in its natural state (Figure 2C). The penile cuticle and surrounding epidermis were thin and folded (Figure 2F), conferring flexibility. The ventral side and base were densely clustered with elongated setae (Figure 3E,G).

### 3.2. Composition and Morphology of the Internal Reproductive System

**Figure 4:**
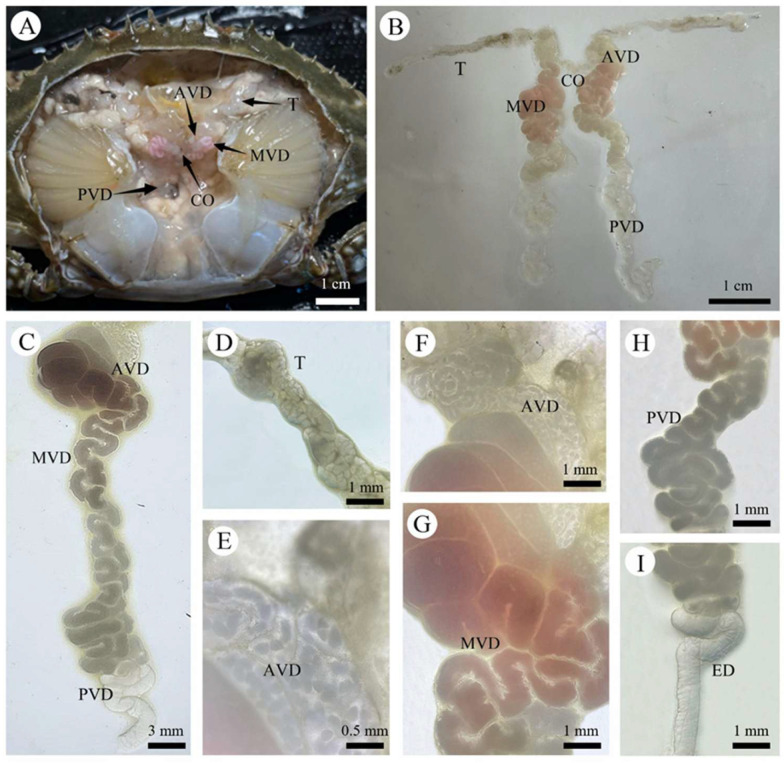
Internal Reproductive System of the Male *P. trituberculatus*. (**A**) Gross anatomy of the internal male reproductive system. (**B**) Composition of the internal male reproductive system. (**C**) Vas deferens. (**D**) Testis. (**E**) Micrograph of spermatophores within the anterior vas deferens (AVD). (**F**) Anterior vas deferens (AVD). (**G**) Middle vas deferens (MVD). (**H**) Posterior vas deferens (PVD). (**I**) Ejaculatory duct (ED). AVD: anterior vas deferens; MVD: middle vas deferens; PVD: posterior vas deferens; T: testis; CO: transverse commissure; ED: ejaculatory duct.

The male reproductive system of *P. trituberculatus* exhibited a bilaterally symmetrical, H-shaped configuration. It consisted of paired testes, paired VD with distinct regional differentiations, and paired ejaculatory ducts. The system was located in the anteromedial portion of the cephalothoracic cavity, dorsal to the hepatopancreas (Figure 4A). The entire reproductive system was enveloped by a layer of connective tissue, which connected it to other components within the body cavity.

#### 3.2.1. Testes (T)

The testes are key components of the male reproductive system in *P. trituberculatus*. They appeared as translucent, ribbon-like organs composed of multiple highly convoluted seminiferous tubules and exhibited a soft texture. Located in the anterior part of the cephalothoracic cavity, these tubular organs extended in a splayed configuration along both sides of the stomach and were connected by a transverse commissure between the posterior stomach and the anterior region of the heart (Figure 4A,B). Their length is typically correlated with crab body size. In the juvenile stage, the testes were relatively small and indistinct, presenting as slender, filamentous structures. As *P. trituberculatus* grew and developed, the testes gradually increased in size, became more turgid, and were filled with abundant germ cells (Figure 4D).

#### 3.2.2. Vas Deferens (VD)

The VD consisted of a pair of elongated and highly coiled tubules extending longitudinally within the anteromedial part of the body. They served as crucial conduits connecting the testes to the ejaculatory ducts. On the basis of differences in tubule diameter, histological structure, and function, the VD is conventionally divided into three distinct regions: the anterior vas deferens (AVD), the middle vas deferens (MVD), and the posterior vas deferens (PVD) (Figure 4C).

#### 3.2.3. Anterior Vas Deferens (AVD)

The anterior section of the VD, which connects to the testes, was a delicate, translucent, and highly convoluted structure with a flocculent appearance (Figure 4F). The AVD is the site where the primary spermatophore wall is formed and the spermatophores are assembled (Figure 4E). Following their formation, the spermatophores are transferred to the middle Vas deferens (MVD), where they become enveloped and stored in seminal fluid. The wall of the AVD consisted of an outer connective tissue layer and a middle muscular layer.

#### 3.2.4. Median Vas Deferens (MVD)

The middle vas deferens was the widest segment of the duct and appeared as a coiled, tubular structure with pink and white coloration (Figure 4G). This region is responsible for producing the majority of the seminal fluid and for storing the spermatophores transferred from the AVD. The secondary spermatophore wall is also formed within the MVD. Its wall was slightly thicker than that of the AVD but shared a similar histological organization, comprising an outer connective tissue layer and a middle muscular layer.

#### 3.2.5. Posterior Vas Deferens (PVD)

The posterior vas deferens presented as a translucent, tubular structure (Figure 4H). Its wall gradually tapered to connect with the ejaculatory duct. The histological structure of the wall was similar to that of the anterior and middle regions, consisting of an outer connective tissue layer and a middle muscular layer. No spermatophores were observed within the PVD.

#### 3.2.6. Ejaculatory Duct(ED)

The ejaculatory duct represented the terminal portion of the male reproductive system. It was relatively short in length and exhibited a uniform diameter, presenting as a smooth, straight, tubular structure (Figure 4I). The duct terminated at an opening located between the basal musculature of the swimming leg (pereiopod 5) and the gonopore (Figure 2A), where it connected to the penis.

## 4. Discussion

### 4.1. Structural and Functional Characteristics of G1 and Its Interspecific Comparison

The male reproductive system of *P. trituberculatus* has an external copulatory apparatus and internal organs, including the testes, VD, and ejaculatory ducts. The external copulatory system primarily consists of the first gonopod (G1), the second gonopod (G2), and the penis, which is the terminal extension of the ejaculatory duct. During copulation, the male crab secures the female beneath its abdomen using its pereiopods. After the female molts, the male turns her over, leading to the opening of both abdomens. The male then inserts the G1 into the female’s gonophore to transfer spermatophores via the penis and gonopods into the female’s spermatheca [30,31]. This study revealed that the G1 in *P. trituberculatus* is tubular, forming a hollow conduit for spermatophore transfer through the infolding of its inner wall. The pinhole-like opening at the apex of G1, along with two rows of robust, downward-projecting spines on its lateral sides, anchors the female gonopore to prevent disengagement during copulation. The pronounced outward curvature of the G1’s distal end appears to be an adaptation to the abdominal angle of the inverted female, facilitating successful insertion and seminal transfer. In addition, the spines distributed along the upper one-third of the G1 may correspond structurally to the morphology of the female gonopore. Functionally, the G1 of *P. trituberculatus* is analogous to that of the mud crab *Scylla serrata* (Forskål, 1775), yet it is structurally distinct. The G1 of *S. serrata* features a laterally flattened apex with a unique nozzle-like structure; this syringe-needle-like morphology potentially reduces resistance during insertion into the female’s spermatheca. The spinules are densely distributed over the anterior surface and the curved region, where their high density may assist in controlling the depth of penetration into the spermatheca. Similarly, the posteriorly oriented projection enhances stability and prevents its dislodgement within the female genital tract during copulation [9,10].

### 4.2. Morphological Differentiation of G2 and Its Pumping Function Mechanism

Male crabs across different taxa in the Brachyura have evolved highly specialized gonopods. However, their specific morphological configurations and functional mechanisms significantly differ. For instance, in species of the family *Calappidae*, such as *Calappa pelii* (Herbst, 1801) and *Calappula saussurei* (Rathbun, 1898), the second gonopod (G2) is significantly longer than the first gonopod (G1). This elongated G2 is inserted into the female spermatheca, enabling the precise delivery of spermatophores to a specific storage compartment [12]. Nevertheless, the second gonopod (G2) in the snow crab is markedly short and features a distinct “annular ridge” at its apex. *Cardisoma guanhumi* [13]. In the present study, the second gonopod (G2) of *P. trituberculatus* exhibited a fish-mouth-like morphology with a closed apex. Its upper portion displays a columnar structure, and its overall size is approximately one-fifth that of the G1. During copulation, the G2 acts as a piston in the G1, using a pumping action to propel the spermatophore in coordination with the penis. The elongated, slit-like folds on the G2 surface may function to reduce frictional resistance during the propulsion of the sperm mass, thereby enhancing the efficiency of spermatophore displacement. Furthermore, the plumose setae surrounding the basal opening of the G1 minimizes friction between the G1 and G2 during this pumping motion while simultaneously helping to prevent leakage of the spermatophores during transfer. Although the G2 of *P. trituberculatus* is short, its fish-mouth-shaped terminus, adorned with surface folds and blunt teeth, may serve dual functions of guidance and sealing. The mating strategy relies on the synergy between the tubular G1 and piston-like G2. The pumping action is compelled by the subtle abdominal contractions of males, which move the G2 reciprocally within the G1 to push spermatophores toward its apical opening and into the female’s spermatheca [6,7,13].

### 4.3. Morphological Adaptability of the Penis and Evolutionary Commonality of Reproductive Mechanisms

The penis length of *P. trituberculatus* ranged from 0.4 to 0.8 cm, a dimension adapted to the size of the basal opening of the first gonopod (G1). Its inherent flexibility enables smooth insertion into the G1 basal aperture during copulatory activity, thus promoting efficient spermatophore transfer. Moreover, the setae distributed on the ventral aspect and base of the penis enhance the seal at the connection point and prevent the entry of external particulate contaminants. Notably, the integrated pumping and transfer mechanism involving the coordinated operation of the G1, G2, and penis is not unique to *P. trituberculatus*. Researchers have also reported this mechanism in taxonomically diverse brachyuran groups, including European pea crabs, snow crabs, and freshwater crabs of the genus *Potamon*. This widespread phylogenetic distribution provides evidence for an evolutionary trend toward the functional integration of pumping apparatuses across multiple crab lineages.

### 4.4. Structural Function and Evolutionary Differentiation of the Internal Reproductive System

The reproductive system of *P. trituberculatus* has a bilaterally symmetric, “H”-shaped configuration. Specifically, the testes are located in the anterodorsal region of the cephalothorax, with the left and right lobes connected by a transverse commissure. Additionally, the VD is composed of three distinct segments, the AVD, MVD, and PVD, that connect to the penis through the ejaculatory duct. At the terminal end of the ejaculatory duct is a penile papilla located centrally on the lateral aspect of the coxa of the last pair of pereiopods. In the anterior vas deferens (AVD) of *P. trituberculatus*, dense acidophilic structural proteins constitute the primary spermatophore wall and facilitate spermatophore formation. Simultaneously, a moderately dense polysaccharide–protein matrix is secreted to embed the sperm mass. This structural and biochemical pattern of spermatophore formation has also been reported in other brachyuran species, such as the blue land crab (*Cardisoma guanhumi*) and the ornate blue crab *(Callinectes ornatus*). This finding indicates its prevalence across taxa [19,20,32]. The lumen of the median vas deferens (MVD) is markedly dilated into glandular sacs or folds to increase the surface area. This segment produces most of the seminal fluid, stores the spermatophores and forms its secondary wall. Consequently, spermatophore maturation is a continuous process in which the functions of the AVD and MVD are closely interconnected. Moreover, the structural differences between these two segments directly reflect distinct stages of spermatogenesis. Each region’s architecture corresponds to specific phases of sperm development and packaging [15]. In contrast to the AVD and MVD, no spermatophores were observed in the posterior vas deferens (PVD), although this segment may regulate the speed of spermatophores during passage. Its gradually tapering wall generates pressure that helps propel spermatophores toward the ejaculatory duct, thus potentially facilitating their discharge. The testes of brachyuran species are primarily categorized into two morphological types: tubular and lobular. Simeó et al. described the testes of the short-fingered crab (*Maja brachydactyla*) (Balss, 1922) as tubular, a structure distinctive to only a minority of brachyuran taxa. For example, the testes of *M. brachydactyla* are divided into diverse functional zones (germinal, transformational and evacuation), a histological pattern that differs from the multilobular parallel arrangement found in *P. trituberculatus* [33,34]. Additionally, the testes of most brachyuran species, including *P. trituberculatus*, exhibit a lobular structure characterized by convoluted seminiferous tubules interspersed between the lobules. This lobular testicular structure represents the predominant morphological type among brachyurans. The synchronized and regionalized pattern of spermatogenesis may indicate both functional convergence and divergence compared to species possessing the tubular type. Future research should focus on a comparative analysis of these two architectural forms, particularly regarding their potential differences in terms of spermiogenic efficiency and reproductive output. The number of VD segments varies considerably among brachyuran species, mostly depending on the criteria applied for classification. For instance, the red mangrove root crab (*Goniopsis cruentata*) (Latreille, 1803) has a two-segmented VD, the slipper lobster (*Scyllarus chacei*) (Holthuis, 1960) has four and the hermit crab (*Diogenes pugilator*) (Roux, 1829) has eight. In brachyuran species, morphological and functional variation reflects adaptations to ecological specialization and diverse reproductive strategies [16,35,36].

## 5. Conclusions

This study systematically clarified the morphological characteristics and functional mechanisms of the male reproductive system in *P. trituberculatus* using anatomical, stereomicroscopic, and scanning electron microscopic techniques, filling the gap in the research on the ultrastructure of its copulatory system; its external copulatory system consists of the G1, G2, and the penis, where the tubular G1 with lateral spines can stably anchor the female gonopore during copulation, the piston-like G2 (approximately one-fifth the length of G1) propels spermatophores through pumping movements, and the flexible penis with setae ensures the efficient transfer and sealing of spermatophores, while the internal reproductive system exhibits a bilaterally symmetrical “H”-shaped structure, including testes, a three-segmented vas deferens (AVD, MVD, PVD), and ejaculatory ducts—with the AVD responsible for spermatophore formation, the MVD undertaking seminal fluid secretion and mature spermatophore storage, and the PVD regulating spermatophore transport, collectively completing the process of spermatophore formation, storage, and delivery; these findings not only reveal the adaptive characteristics of the male reproductive system of *P. trituberculatus* to its mating strategy, providing key insights for understanding the evolutionary convergence and divergence of reproductive structures in brachyuran crustaceans, but also clarify the morphological compatibility and functional coordination among reproductive organs.

## Data Availability

The raw data supporting the conclusions of this article will be made available by the authors on request.
